# The influence mechanism of parental emotional companionship on children’s second language acquisition

**DOI:** 10.3389/fpsyg.2022.1034867

**Published:** 2023-01-13

**Authors:** Xiaoxia Cheng, Shike Zhou

**Affiliations:** ^1^School of Educational Sciences, Nanjing Normal University, Nanjing, China; ^2^Jiangsu Institute of Educational Science Research, Nanjing, China

**Keywords:** parental emotional companionship, second language acquisition, internal learning motivation, further study motivation, learning confidence

## Abstract

It has become a consensus that parental emotional companionship can promote the healthy growth of children. However, the theoretical circle still knows little about the relationship between parental emotional companionship and children’s second language acquisition and the internal processes. In this study, the path analysis method was adopted to analyze the academic quality testing data of Grade 5 and Grade 9 students obtained by questionnaire survey method in Jiangsu Province in 2020, so as to explore the influence mechanism of parental emotional companionship on children’s second language acquisition. The results show that parental emotional companionship promotes second language acquisition. Learning confidence and internal learning motivation play an intermediary role in this relationship. Learning confidence positively influences internal learning motivation and plays a chain mediating role. The indirect effect of internal learning motivation in the middle school group is the masking effect. The conclusion of this study reveals the influence mechanism of parental emotional companionship on children’s second language acquisition, which enriches and deepens the theoretical understanding of the affective factors affecting second language acquisition. Theoretical and practical implications, along with limitations and future research directions were discussed.

## Introduction

The General Offices of the CPC Central Committee and The State Council issued the Opinions on Further Reducing the Burden of Homework and After-school Training for Students in Compulsory Education, with a view to reducing the burden of homework and after-school training for primary and middle school students in China. Therefore, primary and middle school students can have more free activity time and parent–child interaction time. Under the background of globalization, international exchanges are increasing day by day in all walks of life. Students’ second language learning promotes personal socialization and contributes to international communication. In addition, the second language learning has also been shown to help individual cognitive development, for example, bilingual need to constantly switch between the two languages, when said a language inhibition of another, it is said that they have an enhanced inhibitory control mechanism, this can make them in other non-verbal cognitive tasks in regulating and control their attention (Adesope et al., 2010; Bialystok et al., 2016). Under the background of double subtraction, the time and energy that students devote to second language learning are shortened and the parent–child interaction time is prolonged. However, many parents do not treat their children’s second language learning in the right way when spending time with their children. Under the new background, what kind of parent–child interaction is beneficial to children’s second language acquisition and its internal mechanism has become a topic worthy of research. This subject has important times value and social value.

Article 17 of the Law of the People’s Republic of China on the Promotion of Family Education, adopted in October 2020, stipulates that parents or other guardians of minors shall reasonably use methods in the implementation of family education, including strengthening parent–child companionship, attaching equal importance to care and strict requirements, and respecting, understanding and encouraging them. It is enough to show that parent–child companionship and emotional companionship based on love, respect, understanding, and encouragement play an important role in promoting the growth of minors.

The subject of this study considers parental emotional companionship as one of the antecedents of children’s second language acquisition, so this study reviews such literature, which shows that most studies focus on learners’ cognitive factors (Semaan and Yamazaki, 2015), learners’ own emotional factors (Xie and Wang, 2022), and school teaching (Sadikoglu and Oktay, 2018; Bai and Robinson, 2022) on second language acquisition. As an indirect influencing factor, parental factors seem to be irrelevant and easy to be ignored. Few studies have looked at the impact of parental companionship on academic performance in specific subjects (Dearing et al., 2004; Englund et al., 2004), only a few studies focused on the differences in the influence of family preconditions on academic achievement in different subjects. For example, studies have found that Chinese students rely more heavily on their family background in second language acquisition than on other subjects such as Chinese and mathematics. By analyzing China’s large-scale survey data (CEPS tracking data), Fang and Huang (2018) prove that economic input affected by family background has a particularly significant impact on urban students’ second language learning. This means that interdisciplinary research may obscure the patterns of learning in particular disciplines (Arens and Jude, 2017). Teachers and parents should adopt different behaviors and attitudes toward students according to the characteristics of different subjects (Régner et al., 2009). In addition, there may be significant differences in the relationship between parental involvement style and academic achievement in specific subjects under different ethnic and cultural backgrounds (Jeynes, 2010). In view of the greater dependence of second language acquisition on family factors and the possible influence of cultural background on this relationship, it is very necessary to study the influence of parents on their children’s second language acquisition under the background of Chinese culture. Among the few literatures on parents’ influence on their children’s second language acquisition, most focus on such issues as parents’ English level, participation in school activities (Jung and Zhang, 2016), expectation level (Suárez-Orozco and Suárez-Orozco, 2001; Cheng and Starks, 2002), Creating a language environment (Goldenberg et al., 2008). Few studies focused on the antecedent cause of parental emotional companionship, and most of them were small sample studies.

In view of the above policy background, practical background, and academic background, this study will use large-scale survey data to explore the impact of parental emotional companionship on children’s second language acquisition and its internal influencing mechanism.

## Literature review and hypothesis

### Theoretical basis

In the second language learning model of Krashen (1981), the “affective filtering hypothesis” holds that learners’ emotional states or attitudes, such as motivation and self-confidence, can affect the comprehensibility input of language learners. According to this model, learners’ good emotional state positively influences their second language learning results. Maslow’s hierarchy of needs theory puts forward five types of needs: physiological needs, safety needs, social needs (belonging and love needs), respect needs, and self-actualization needs. These needs are not only hierarchical but also sequential (Maslow, 1987). Parental emotional companionship satisfies children’s social needs and respect needs, and satisfies the higher level needs. According to Maslow’s hierarchy of needs theory, parents satisfy their children’s emotional needs which are closer to the need of self-actualization through emotional companionship, which is conducive to children’s pursuit of self-worth. As a way of self-actualization, second language learning performance is more likely to be valued by children whose emotional needs are met. Parents adopt an encouraging and supportive attitude in the interaction with their children, timely relieve their children’s bad emotions, and keep learners in a good emotional state, which may improve their children’s second language learning effect. Based on this theory, the following hypotheses are proposed:

*H1*: Parental emotional companionship promotes children’s second language acquisition.

The “affective filtering hypothesis” in Krashen’s (1981) second language learning model focuses on the role of learners’ emotional factors. This study focuses on external emotional factors closely related to learners’ emotional factors. Therefore, this study introduces the cognitive-motivation model theory, which believes that self-cognition and motivation factors mediate the influence of emotional factors on academic achievement (Pekrun, 1992). Among them, the most important mediating factors include students’ learning motivation and learning self-regulation, and positive emotions help to enhance learning motivation (Pekrun et al., 2010). Self-regulation of learning means planning, monitoring, and evaluating one’s own learning in a flexible way, and adjusting learning strategies to meet task demands and progress in the process. Positive emotions contribute to self-regulation of learning (Pekrun et al., 2010). According to this theory, second language learning performance, as a kind of academic achievement, may be influenced by emotional companionship from parents, and motivational factors may play a mediating role in this relationship. Learning self-confidence is an individual’s positive judgment of his ability to complete learning tasks, a positive cognition and evaluation of learning ability, and an important aspect of self-cognition, so learning self-confidence may also mediate this relationship.

### Learning confidence

By discussing school activities with their children, parents can understand their difficulties and provide necessary emotional support. Marsh and Craven (2006) showed that the behavior of parents discussing school activities with their children can improve their children’s academic self-concept, namely the cognition of their own abilities. Such good cognition of self-ability is confidence in learning, and the emotional support of parents conveyed through discussion activities may enhance their children’s learning confidence. Other studies have found that girls will discuss school activities with their parents more, have greater advantages in second language learning, and have higher self-concept in second language subjects. It can be seen that the cognition of their second language learning ability can help learners form learning advantages. Therefore, it can be inferred that parental emotional companionship may promote children’s second language acquisition by improving their learning self-confidence. Arens and Jude's (2017) study demonstrated that students’ academic self-concept (perception of self-competence) mediates the relationship between family interaction and academic achievement in second language. The family interaction in this view can reflect the emotional companionship of parents, which has some similarities with the inference of this study. In addition, according to the cognitive-motivation model theory, learning self-confidence may mediate the influence of emotional factors on academic achievement. Accordingly, the following hypothesis is proposed:

*H2a*: Parents’ emotional companionship enhances their children’s learning confidence;

*H2b*: Parental emotional companionship promotes second language acquisition by enhancing learning confidence.

### Internal learning motivation

Deci and Ryan (1985) believe that intrinsic motivation originates from learners’ interest in the task itself and is the positive emotional experience generated by learners in the process of completing the task, which is a powerful factor to maintain motivation. The greatest common denominator of human beings is their dependence on emotion. Adequate emotional companionship provided by parents can enhance children’s secure attachment and make children more satisfied with their parent–child relationship. Butler (2015) showed in her research that children’s secure attachment to their parents and relationship satisfaction could enhance their internal learning motivation. Therefore, it can be inferred that parents’ emotional companionship may improve their children’s internal motivation in second language learning. Parents are the main factors influencing children’s learning motivation (Butler, 2015). The “affective filtering hypothesis” in Krashen’s (1981) second language learning model shows that motivation is an important factor affecting second language learning. Rumbaut’s (1994) research also showed that some students who had few opportunities to speak a foreign language at home did well in English as a foreign language art class. Their motivation is an important factor in overcoming adverse circumstances and achieving higher academic performance in second language (Fuligni, 1997). Based on the above analysis, it can be concluded that parents’ emotional companionship may improve the second language learning effect by improving their children’s internal learning motivation. Cognitive-motivation model theory believes that motivation factors mediate the influence of emotional factors on academic achievement, which is fully supported by the theory. Accordingly, the following hypothesis is proposed:

*H3a*: Parental emotional companionship enhances children’s internal learning motivation;

*H3b*: Parental emotional companionship promotes second language acquisition by enhancing internal learning motivation.

### Learning confidence and internal learning motivation

Shih (2005) showed that in the process of foreign language learning, the higher confidence children have in language skills, the higher their choice of goals, and the stronger their interest in language learning. This indicates that students’ confidence in second language learning may improve their internal learning motivation. Dörnyei (2009) and Dörnyei and Ushioda (2011) proposed that when completing a certain behavior, we constantly evaluate whether the behavior meets certain conditions and estimate the feasibility of completing the task. The resulting emotional experience will also affect the motivational behavior. In the process of second language learning, students may constantly evaluate their own language learning ability. Positive evaluation of second language learning ability indicates that students have high confidence in this aspect; otherwise, it is low. Such emotional experience in turn affects students’ motivation for second language learning. Based on the above analysis, students’ confidence may improve students’ motivation. Combining this inference with the above hypothesis, it can be inferred that learning self-confidence may play a chain mediating role between parental emotional companionship and second language acquisition outcomes by improving internal learning motivation. Accordingly, the following hypothesis is proposed:

*H4a*: Learning self-confidence positively affects internal learning motivation;

*H4b*: Learning confidence plays a chain mediating role between parents’ emotional companionship and children’s second language acquisition by positively influencing internal learning motivation.

**Figure 1:**
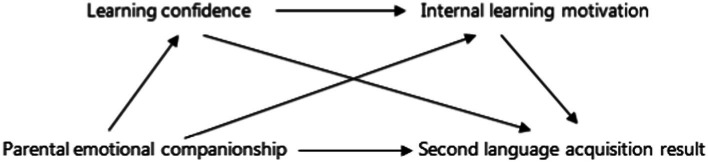
Theoretical model diagram.

Based on the above theoretical basis and literature analysis, this study puts forward the theoretical model in Figure 1, and makes an exploratory analysis of the model based on the provincial survey data of Jiangsu Province in 2020.

## Research method

The data of this study come from the monitoring of the academic quality of basic education in Jiangsu Province in 2020, which is a cooperative project between Provincial Institute of Education and Science and a normal university. By adopting stratified sampling method, 204,205 students in grade 5 and 147,805 students in grade 9 are selected for questionnaire survey and academic level assessment. The data have two advantages: First, the sample size is large to ensure that the data results have the characteristics of a large group. Second, the provincial Institute of Education and Science and normal university professors cooperate in the investigation, and the quality of the data is guaranteed. The survey was approved by the Education Bureau and supported by parents. The questionnaire was filled out in class anonymously, and each group’s advisor had received special training. The survey collected all the data needed for the project of cooperation between the Normal University and the Provincial Institute of Education and Science, covering a wide range of information related to schools, teachers, families, and students. By matching and sorting the data and removing invalid data with missing key information or anomalies, the study obtained valid data for 140,576 fifth-grade students and 100,156 ninth-grade students. Finally, based on the theoretical basis, path analysis is conducted on the independent variables involved in this study (parents’ emotional companionship), intermediary variables (learning confidence and internal learning motivation), and dependent variables (children’s second language acquisition level).

### Participants

The participants of the whole project include researchers of the provincial Institute of Education and Science, professors and graduate students of a normal university, school leaders, teachers, and students, etc. This study focused on the impact of parental emotional presence on second language acquisition. Participants included researchers who organized the project survey and 204,205 students in grade 5 and 147,805 students in grade 9. The Parental Emotional Companionship Questionnaire, filled out by students, measures the degree of emotional companionship perceived by children.

### Instruments

In addition to the basic information of the students, the five-point Likert scale was used for the survey, ranging from strongly disagree (1) to strongly agree (5). The questionnaire was written in Chinese, the first language of the participants.

The proxy variable of the dependent variable: The dependent variable is the final test score of the survey as the proxy variable. This test paper is unified and standardized, which can effectively measure students’ second language learning level.

The scale of Independent variable: The independent variable of this study is parental emotional companionship, which is to investigate the degree of students’ perceived emotional companionship from the perspective of students. The scale developed by Feng (2011) was adopted. There were three questions, and the questions were scored using Likert’s five-point scale. Example item: “When things go wrong, I can feel my parents trying to encourage and comfort me” (1 = strongly disagree, 5 = strongly agree).

The scales of intermediate variables: The scales of learning confidence and internal learning motivation were developed by the provincial test project team according to relevant theories, and strict reliability and validity tests were carried out. There are five items in each scale. Five-point Likert scale was used for scoring. The example item of learning confidence is “If a problem seems too complicated, I do not want to try it (reverse course).” The example item of internal learning motivation is “as long as I can learn knowledge, difficult learning content or topics are also attractive to me” (1 = strongly disagree, 5 = strongly agree).

### Data analysis

After the survey data were obtained, the mean values of parental emotional companionship, learning confidence, and internal learning motivation were calculated using SPSS software. Then, all variables were standardized by SPSS. Finally, the PROCESS 3.5 program was embedded in SPSS, and the mediation model 6 which was consistent with the theoretical model of this study was selected. Through the Bootstrap method, the regression results between variables and the mediation effect index were obtained by repeated sampling 5,000 times within the 95% confidence interval.

## Research results

### Descriptive statistics

Table 1 shows the descriptive analysis results of variables, including the maximum value, minimum value, average value, standard deviation of each variable, and sample size, showing the basic information of the samples, among which the more important information is that parents’ emotional companionship, students’ learning confidence, and internal learning motivation in primary school are all higher than those in middle school, which lays the foundation for the subsequent test of main effect and mediating effect.

**Table 1:** Descriptive statistics of data.

Variable name	Primary school				Middle school			
	Minimum value	Maximum value	Average value	SD	Minimum value	Maximum value	Average value	SD
Second language acquisition results	232.1	636.98	510.10	68.41	227.24	707.05	516.35	75.80
Parental emotional companionship	1	5	4.28	0.93	1	5	3.49	1.10
Learning confidence	1	5	4.46	0.72	1	5	3.94	0.84
Internal learning motivation	1	5	4.56	0.74	1	5	3.36	0.67
Number of active cases	140,576				100,156			

### Scale reliability and validity test

In this study, Cronbach’s *α* value and combined reliability CR values were used to measure the reliability of the scale. The results in Table 2 show that the Cronbach’s *α* values are all greater than 0.8, and the combined reliability CR values are all greater than 0.7, indicating that the internal consistency reliability of variables is relatively ideal. Meanwhile, the average extraction variance value (AVE) was used to measure the scale validity. AVE values were all greater than 0.5, which proved that the scale had good internal validity. In addition, the factor loading of all the questions is greater than 0.6, CIF values are all greater than 0.92, the RMSEA values were all less than 0.06, and the model fit was good.

**Table 2:** Reliability and validity test results of variables.

Variable name	Item of measurement	Cronbach’s *α*	AVE	CR
Parental emotional companionship	My parents often discuss things with me about school.	0.877/0.887	0.545/0.611	0.712/0.756
	When things are not going well, I can feel that my parents are trying to encourage me and comfort me.			
	I share my secrets and personal feelings with my parents.			
Learning confidence	If I work hard, I can overcome the difficulties in my study.	0.801/0.837	0.501/0.512	0.797/0.836
	If a problem seems complicated, I do not want to try it. (reverse)			
	I’m not cut out for learning. (reverse)			
	I’m sure I can do well in the exam.			
	I think the goals I set for myself are generally achieved.			
Internal learning motivation	Reading learning books is a pleasure.	0.859/0.884	0.558/0.614	0.862/0.887
	I often experience a kind of happiness in the process of learning.			
	As long as I can learn knowledge, difficult learning content or topics are also attractive to me.			
	I like to study, so I will study hard.			
	I hope I can make some achievements in my study in the future.			

The first numbers in the table belong to the primary school group, and the last numbers belong to the middle school group.

### Main effect and mediating effect test

In order to examine the subjective effect of parental emotional companionship in promoting children’s second language acquisition and the mediating effect between learning confidence and internal learning motivation, the Bootstrap method was adopted. PROCESS 3.5 program was embedded in SPSS, 95% confidence interval was selected, and sampling was repeated 5,000 times. Tables 3, 4 report the fitting indexes and regression coefficients of the path model of the primary school group and the middle school group. According to the regression results, the *p* values were all less than 0.001, and the 95% confidence interval did not contain 0, indicating a good model fitting. Tables 5, 6 report the results of mediating effect size and proportion.

**Table 3:** Regression results of variables in primary school group.

Outcome variable	Predictive variable	*R* ^2^	*F*	*β*	SE	*t*	LLCI	ULCI
Learning confidence	Parental emotional companionship	0.213^***^	37288.4	0.603^***^	0.003	193.1	0.597	0.609
Internal learning motivation	Parental emotional companionship	0.762^***^	221269.6	0.089^***^	0.002	45.8	0.085	0.093
	Learning confidence			0.841^***^	0.001	567.7	0.838	0.844
Second language acquisition results	Parental emotional companionship	0.074^***^	3687.7	0.117^***^	0.004	30.5	0.110	0.124
	Learning confidence			0.105^***^	0.005	19.7	0.094	0.115
	Internal learning motivation			0.120^***^	0.005	22.7	0.110	0.131
Second language acquisition results	Parental emotional companionship (direct effect)	0.037^***^	5343.2	0.252^***^	0.003	73.1	0.245	0.259

***p < 0.001.

**Table 4:** Regression results of each variable in the middle school group.

Outcome variable	Predictive variable	*R* ^2^	*F*	*β*	SE	*t*	LLCI	ULCI
Learning confidence	Parental emotional companionship	0.209^***^	26491.7	0.348^***^	0.0021	162.8	0.344	0.352
Internal learning motivation	Parental emotional companionship	0.349^***^	26793.5	0.163^***^	0.0018	92.9	0.16	0.166
	Learning confidence			0.337^***^	0.0023	146.1	0.333	0.342
Second language acquisition results	Parental emotional companionship	0.073^***^	2638.4	0.01^***^	0.0005	20.3	0.009	0.011
	Learning confidence			0.044^***^	0.0007	62.3	0.043	0.046
	Internal learning motivation			−0.001	0.0009	−2.14	−0.004	0.000
Second language acquisition results	Parental emotional companionship(direct effect)	0.031^***^	3195.5	0.025^***^	0.0004	56.5	0.024	0.026

^***^p < 0.001.

**Table 5:** Results of Bootstrap test of mediating effect in primary school group.

	Effect	Boot SE	Boot LLCI	Boot ULCI	Ratio of indirect to total effect	Ratio of indirect to direct effect
Total effect	0.252	0.003	0.245	0.259		
Direct effect	0.117	0.004	0.11	0.125		
Total indirect effect	0.135	0.002	0.131	0.139	53.57%	115.38%
Parental emotional companionship–Learning confidence–Second language acquisition	0.063	0.003	0.057	0.07	25.00%	53.85%
Parental emotional companionship–Internal learning motivation–Second language acquisition	0.011	0.0006	0.01	0.012	4.37%	9.40%
Parental emotional companionship–Learning confidence–Internal learning motivation–Second language acquisition	0.061	0.003	0.055	0.067	24.21%	52.14%

**Table 6:** Results of Bootstrap test of mediating effect in middle school group.

	Effect	Boot SE	Boot LLCI	Boot ULCI	Ratio of indirect to total effect	Ratio of indirect to direct effect
Total effect	0.0253	0.0004	0.0244	0.0262		
Direct effect	0.0104	0.0005	0.0094	0.0114		
Total indirect effect	0.0149	0.0003	0.0143	0.0155	58.89%	143.27%
Parental emotional companionship–Learning confidence–Second language acquisition	0.0154	0.0003	0.0149	0.016	60.87%	148.08%
Parental emotional companionship–Internal learning motivation–Second language acquisition	−0.0003	0.0002	−0.0006	0		2.88%
Parental emotional companionship–Learning confidence–Internal learning motivation–Second language acquisition	−0.0002	0.0001	−0.0004	0		

Hypothesis 1 focuses on the direct impact of parental emotional companionship on second language acquisition. As can be seen from Tables 3, 4, the direct effect of parental emotional companionship on second language acquisition of children in primary school group is significant (*β* = 0.252, *p* < 0.001), the direct effect was also significant in the middle school group (*β* = 0.025, *p* < 0.001). And the results of both groups showed that the 95% confidence interval did not contain 0, which further verified the stability of the results. Hypothesis 1 is verified by empirical results.

Hypothesis 2a focuses on the effect of parental emotional presence on children’s academic confidence. As can be seen from Tables 3, 4, the influence of parents’ emotional companionship on their children’s learning self-confidence is significant in the primary school group (*β* = 0.603, *p* < 0.001), the effect was also significant in the middle school group (*β* = 0.348, *p* < 0.001). And the results of both groups showed that the 95% confidence interval did not contain 0, which further verified the stability of the results. Hypothesis 2a is verified by empirical results.

Hypothesis 3a focuses on the influence of parental emotional companionship on children’s internal learning motivation. As can be seen from Tables 3, 4, the influence of parents’ emotional companionship on children’s internal learning motivation in primary school group is significant (*β* = 0.089, *p* < 0.001), the effect was also significant in the middle school group (*β* = 0.163, *p* < 0.001). And the results of both groups showed that the 95% confidence interval did not contain 0, which further verified the stability of the results. Hypothesis 3a is verified by empirical results.

Hypothesis 4a focuses on the influence of learning confidence on internal learning motivation. As can be seen from Tables 3, 4, the influence of learning self-confidence on internal learning motivation of primary school children is significant (*β* = 0.841, *p* < 0.001), the effect was also significant in the middle school group (*β* = 0.337, *p* < 0.001). And the results of both groups showed that the 95% confidence interval did not contain 0, which further verified the stability of the results. Hypothesis 3a is verified by empirical results.

Hypothesis 2b Focusing on parental emotional companionship promotes second language acquisition by enhancing learning confidence. From Tables 5, 6, as can be seen from Tables 5, 6, in the primary school group, the indirect effect size of learning confidence between parents’ emotional companionship and second language acquisition is 0.063, confidence interval is [0.057, 0.07], excluding 0, and the indirect effect is significant. The indirect effect size of the middle school group was 0.0154, and the confidence interval was [0.0149, 0.016], excluding 0, which was also significant. Hypothesis 2a is verified by empirical results.

Hypothesis 3b focusing on parental emotional companionship promotes children’s second language acquisition by improving internal learning motivation. Tables 5, 6 show that in the primary school group, the indirect effect size of internal learning motivation between parental emotional companionship and second language acquisition is 0.011, confidence interval is [0.01, 0.012], excluding 0, and the indirect effect is significant. Hypothesis 3b has been verified by the empirical results of the primary school group. This indirect effect size was −0.0003 for the middle school group. According to the research results of Wen and Ye (2014), if the mediating effect has the same sign as the total effect, it belongs to partial mediating effect; if the total effect has different sign, it belongs to masking effect. The absolute value of the ratio between indirect effect and direct effect is reported without considering whether the result is significant or not. Therefore, it can be inferred that the internal learning motivation of the middle school group shows a masking effect on the relationship between parents’ emotional companionship and children’s second language acquisition.

Hypothesis 4b focusing on learning confidence positively influences internal learning motivation, which plays a chain mediator role between parents’ emotional companionship and children’s second language acquisition. As can be seen from Tables 5, 6, the chain mediating effect size in primary school group is 0.061, and the confidence interval is [0.055, 0.067], excluding 0, and the chain effect is significant. Influenced by the masking effect of internal learning motivation, the indirect effect size of the middle school group is also negative (−0.0002). The masking effect of internal learning motivation will be discussed in the discussion section combined with relevant theories.

**Figure 2:**
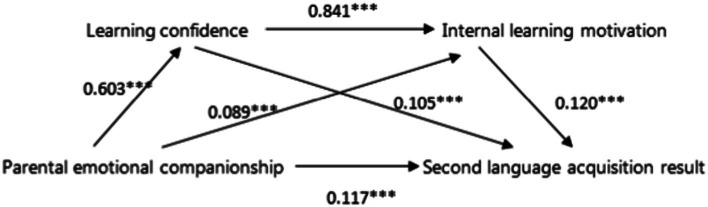
Verification result of primary school group theoretical model.

**Figure 3:**
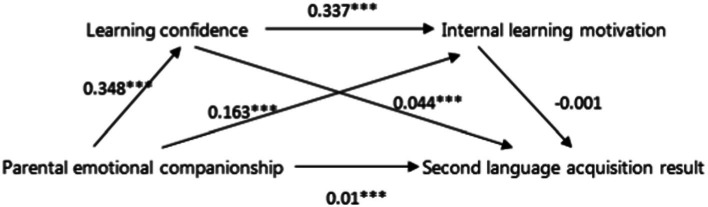
Verification result of the theoretical model of the middle school group.

The assumed theoretical model focuses on whether the influence between variables is significant, whether the mediating effect of learning confidence and internal learning motivation is significant, and whether the chain mediating effect of learning confidence enhancing internal learning motivation is significant between parents’ emotional companionship and children’s second language acquisition. As can be seen in Figure 2, the influence coefficients among all variables in the primary school group are significant. Combined with the test results of the mediation effect mentioned above, the model is established. As shown in Figure 3, in the middle school group, the influence coefficient of internal learning motivation on second language acquisition was not significant, but all the others were significant. The product sign of the influence coefficient of parental emotional companionship to internal learning motivation multiplied by the influence coefficient of internal learning motivation to second language acquisition was opposite to the sign of the influence coefficient of parental emotional companionship to children’s second language acquisition, then the masking effect of internal learning motivation was considered, and the model was still valid.

## Discussion

### Theoretical model

Based on the second language learning model “affective filtering hypothesis,” cognitive-motivation model, and related literature review, this paper constructs a theoretical model of the influence of parental emotional companionship on children’s second language acquisition. Through Bootstrap method, PROCESS 3.5 program was embedded in SPSS to analyze the quality detection data of academic level in Jiangsu Province in 2020, and verify the theoretical model. The results of data analysis support the theoretical model: Learning confidence and internal learning motivation play a mediating role in the relationship between parents’ emotional companionship and children’s second language acquisition. In addition, learning confidence positively influences internal learning motivation, which plays a chain mediating role. Among them, the internal learning motivation of the junior middle school group showed masking effect. On the basis of discussing the theoretical model of parental affective companionship affecting children’s second language acquisition, this study will discuss the masking effect of the junior middle school group based on the internal motivation theory of Desi and Frast.

In terms of the direct impact of parental emotional companionship on children’s second language acquisition, the empirical results of both the elementary and middle school groups are significant, verifying the subject effect of the research hypothesis. In addition, from the perspective of Maslow’s need theory, parents’ emotional companionship can satisfy their children’s needs of belonging, love, and respect, thus helping them to pursue a higher level of self-realization. The influence of parental emotional companionship on second language acquisition as a way of self-actualization is consistent with the idea contained in this theoretical model. Existing studies also provide some support for this study. For example, parents’ emotional support can help second language learners’ academic achievement and literacy ability (Percy et al., 2013; Shin and Seger, 2016). Kim and Lee (2019) proved the importance of teachers’ and parents’ emotional support to children’s English academic achievement through empirical research, and encouraged more emotional support for students. The study of Ham et al. (2020) shows that students’ perception of parental emotional support is higher, and the second language learning effect is better. Students with lower perceived academic support have lower academic performance. These conclusions are consistent with the results of this study, but they are all drawn in the context outside of China. This study shows that under the background of Chinese culture, parents’ emotional companionship still promotes children’s second language acquisition, which brings inspiration to family interaction in China. In addition, these relevant studies did not deeply explore the internal influencing mechanism, and this study goes further in theoretical depth.

In terms of the mediating effect of children’s internal learning motivation and learning confidence, the establishment of this hypothesis is consistent with the cognitive-motivation model which emphasizes that self-cognition and motivation play a mediating role between affective factors and academic achievement. There are few completely consistent results, but they also provide some support. Csizér and Kormos (2009) found that parents’ emotional support was an important factor affecting Hungarian students’ attitude and motivation in second language learning. Morris et al. (2013) also found that in the second language learning of Korean students, parental encouragement is positively correlated with learning motivation, while parental neglect is negatively correlated with motivation. Both studies support the motivational boost of parental emotional companionship. Crookes and Schmidt’s (1991) research shows that higher learning motivation helps foreign language learners to improve their attention while using more cognitive strategies，one of the factors determining motivation is whether personal needs are met. This study emphasizes that demand fulfillment affects learners’ behaviors in the process of foreign language learning by influencing their motivation, which is somewhat related to, but not completely consistent with this study, which focuses specifically on the emotional needs and the outcomes of second language learning rather than behaviors. Crookes and Schmidt’s (1991) study brings some enlightenment to this research: as the closest people of children, parents’ emotional companionship can especially meet their children’s emotional needs, thus promoting the improvement of learning motivation, and then learners will be more focused on their second language learning and use more strategies, thus improving their second language academic level. In addition, according to the emotional security hypothesis, a good parent–child relationship can satisfy the emotional support that primary and secondary school students need for their ability development, thus promoting them to explore the outside world more bravely and openly (Davies and Cummings, 1994). Exploring the outside world bravely and openly is a sign of confidence, and the emotional support from parents provided by a good parent–child relationship can promote children to explore new things confidently. The first language is something everyone is familiar with, while the second language is a new and challenging thing from the outside world for learners. Parents’ emotional support can improve their children’s confidence in exploring the second language world and thus improve their second language learning. The higher the learner’s confidence in second language learning, the more willing they are to learn this language independently, and at the same time, they will challenge higher learning goals. Students’ confidence in second language learning positively promotes the internal learning motivation. Falout and Maruyama (2004) found that impaired self-confidence was the main factor leading to negative motivation. Although the research focused on the opposite direction, it was consistent with the relationship between self-confidence and motivation emphasized in the chain mediation of this study.

As for the masking effect of internal learning motivation in the middle school group, the effect of internal learning motivation on the relationship between parents’ emotional companionship and children’s second language acquisition may be affected by the degree of perceived autonomy of children. The theory of motivation by Desi and Forrester (2021a) shows that supporting and affirming the social environment of people’s perceived autonomy will enhance intrinsic motivation, while weakening that will destroy intrinsic motivation. The research of Ryan and Lynch (1989) found that teenagers express their will under the premise of relying on their parents, rather than strongly demanding to live independently from their parents. Teenagers are more likely to strive for a certain degree of autonomy from the will. In this critical period, if parents’ emotional companionship fails to support their pursuit of autonomy, it will destroy their children’s internal learning motivation and negatively affect their academic achievement. As one of the influential factors of social environment, parental emotional companionship can both enhance and destroy children’s internal learning motivation, which is related to the degree of parental control and autonomy felt by children. Therefore, the masking effect of internal learning motivation may be related to junior middle school students’ pursuit of autonomy and their failure to fully realize it. Desi and Forrester (2021b) argue that people are born to establish emotional connection with others and then rely on others. Dependence is motivated by the need to love and be loved. If we feel autonomous dependence, it is natural, beneficial, and healthy. Therefore, parents should pay attention to the realization of children’s autonomy while providing emotional support for their children. Nikolov (1999) took students aged 6–14 as the research object and studied their motivation for learning a foreign language. The results showed that children aged 11–14 were more likely to be affected by utilitarian factors of language rather than intrinsic motivation. This is consistent with the results of this study. The conclusion that adolescents are less susceptible to internal motivation is consistent with the results of this study. Existentialist philosophy holds that people always have a choice, that people create their own existence through their own choices every moment; therefore, they are fully responsible for themselves, rather than yielding to the forces of chaos and control. Parents should avoid using the power of control while providing emotional support for their children, so that their children can fully feel competent and independent, form a healthy and natural dependence, and promote the improvement of internal learning motivation.

### Theoretical significance

Based on the theory, this study explores the relationship and internal mechanism between parents’ emotional companionship and their children’s second language acquisition, and enhances the understanding of cognitive improvement through emotional flow between parents and children. The “affective filtering hypothesis” in the second language learning model has been widely accepted, which emphasizes the influence of learners’ emotional state on language learning. Existing literature has shown that learning confidence and motivation can effectively promote learners’ second language acquisition, and learners’ emotional factors have attracted much attention. The path by which emotional flow between parents and children promotes cognition is not clear. Therefore, based on the cognitive-motivation model theory, this study explores the influence of parental emotional companionship on learners’ learning-related emotional factors through emotional flow, and then promotes second language acquisition. The theoretical framework of this study deepens the theoretical understanding of the role of parental affective companionship in children’s second language acquisition and broadens the thinking of future research.

### Practical significance

In real life, parents most often choose to directly participate in their children’s learning process to help their children acquire second language, which often leads to parent–child relationship tension and children’s learning anxiety. This study has important guiding significance for parents’ companionship and children’s second language acquisition. On the one hand, in order to promote their children’s second language acquisition, parents should pay attention to emotional companionship and treat their children in an understanding, encouraging, and inclusive way. Only in this way can their children’s learning motivation and learning confidence be enhanced. Through the input of external emotions, the inner emotional problems in second language acquisition can be solved. However, parents should pay attention to fully respect their children’s autonomy and freedom in the process of emotional companionship, and should not turn emotional companionship into emotional shackles. On the other hand, in the process of second language learning, children can turn to their parents for understanding, encouragement, and support if they encounter lack of self-confidence or lack of motivation. Even though parents may not be proficient in a second language, they can still be helpful in terms of emotional stimulation.

### Limitations and future prospects

This study still has some limitations. First, there are few similar studies, so it is difficult to find the same results for comparative demonstration. In future studies, repeated demonstrations in different cultural backgrounds can be considered to further verify the validity of this theoretical model. Second, the data used in this study are panel data, which affects the determination of the stability of the results to a certain extent. In future studies, panel data can be used to verify the validity of the model again.

## Conclusion

Scholars have been devoted to exploring the influencing factors of learners’ second language acquisition, but few studies have focused on the influence of parents’ emotional companionship on children’s second language acquisition. Based on the second language learning model “affective filtering hypothesis,” cognitive-motivation model, and related literature review, this paper constructs a theoretical model of the influence of parental emotional companionship on children’s second language acquisition. Based on the academic quality monitoring data of Jiangsu Province in 2020, the Bootstrap method was used for empirical test. The results showed that parental emotional companionship positively promoted their children’s second language acquisition through the mediating effects of learning confidence and internal learning motivation, and learning confidence affected internal learning motivation and played a chain mediating role between this relationships. The data analysis results in the middle school show certain particularity, and the indirect effect of internal learning motivation is the masking effect. Based on the motivation theory, the mediating effect of internal learning motivation between parents’ emotional companionship and children’s second language acquisition may be influenced by the degree of perceived autonomy of children. Due to the current data, this study can only explain this result from a theoretical perspective. Future studies may consider including the degree of children’s perceived autonomy as a moderating variable between parents’ emotional companionship and children’s internal learning motivation to provide empirical evidence. In addition, the most important thing is that parents should strengthen the emotional accompaniment to their children, while paying attention not to turn emotional accompaniment into emotional shackles, and should fully meet their children’s needs for autonomy.

## Data availability statement

The raw data supporting the conclusions of this article will be made available by the author, without undue reservation.

## Ethics statement

The studies involving human participants were reviewed and approved by Jiangsu Institute of Educational Science Research. Written informed consent to participate in this study was provided by the participants’ legal guardian/next of kin.

## Author contributions

XC: data analysis, article writing, and most of the later revision work were done. SZ: completes the search, analysis and supplement of new literature at the time of revision and proofreads all the revised manuscripts.

## Conflict of interest

The authors declare that the research was conducted in the absence of any commercial or financial relationships that could be construed as a potential conflict of interest.

## Publisher’s note

All claims expressed in this article are solely those of the authors and do not necessarily represent those of their affiliated organizations, or those of the publisher, the editors and the reviewers. Any product that may be evaluated in this article, or claim that may be made by its manufacturer, is not guaranteed or endorsed by the publisher.

## References

[ref1] Adesope O. O. Lavin T. Thompson T. Ungerleider C. (2010). A systematic review and meta-analysis of the cognitive correlates of bilingualism. Rev. Educ. Res. 80, 207–245. doi: 10.3102/0034654310368803

[ref2] Arens K. Jude N. (2017). Parental involvement and student achievement in two language domains: indirect relations and generalizability across migration status. Learn. Individ. Differ. 53, 145–155. doi: 10.1016/j.lindif.2016.12.001

[ref3] Bai G. R. Robinson C. S. (2022). Second language and its significance in teaching and learning through task-based learning: literature review. Int. J. Early Child. Spec. Educ. 14, 712–718. doi: 10.9756/INT-JECSE/V14I1.221083

[ref4] Bialystok E. Abutalebi J. Bak T. H. Burke D. M. Kroll J. F. (2016). Aging in two languages: implications for public health. Ageing Res. Rev. 27, 56–60. doi: 10.1016/j.arr.2016.03.003, PMID: 26993154 PMC4837064

[ref5] Butler Y. G. (2015). Parental factors in children’s motivation for learning English: a case in China. Res. Pap. Educ. 30, 164–191. doi: 10.1080/02671522.2014.891643

[ref6] Cheng S. Starks B. (2002). Racial differences in the effects of significant others on students’educational expectations. Sociol. Educ. 75, 306–327. doi: 10.2307/3090281

[ref7] Crookes G. Schmidt R. W. (1991). Motivation: reopening the research agenda. Lang. Learn. 41, 469–512. doi: 10.1111/j.1467-1770.1991.tb00690.x

[ref8] Csizér K. Kormos J. (2009). “An investigation into the relationship of L2 motivation and cross-cultural contact among elementary school students” in Early Learning of Modern Foreign Languages: Processes and Outcomes. ed. Nikolov M. (Bristol: Multilingual Matters), 62–74.

[ref9] Davies P. T. Cummings E. M. (1994). Marital conflict and child adjustment: an emotional security hypothesis. Psychol. Bull. 116, 387–411. doi: 10.1037/0033-2909.116.3.387, PMID: 7809306

[ref10] Dearing E. McCartney K. Weiss H. B. Kreider H. Simpkins S. (2004). The promotive effect of family educational involvement for low-income children's literacy. J. Sch. Psychol. 42, 445–460. doi: 10.1016/j.jsp.2004.07.002

[ref11] Deci E. L. Ryan R. M. (1985). Intrinsic Motivation and Self-Determination in Human Behavior. New York: Plenum.

[ref12] Desi E. L. Forrester R. (2021a). Intrinsic motivation. China: Machinery Industry Publishing, 82.

[ref13] Desi E. L. Forrester R. (2021b). Intrinsic motivation. China: Machinery Industry Publishing, 92

[ref14] Dörnyei Z. (2009). The Psychology of Second Language Acquisition. Oxford: Oxford University Press.

[ref15] Dörnyei Z. Ushioda E. (2011). Teaching and Researching Motivation, Harlow: Longman.

[ref16] Englund M. M. Luckner A. E. Whaley G. J. L. Egeland B. (2004). Children's achievement in early elementary school: longitudinal effects of parental involvement, expectations, and quality of assistance. J. Educ. Psychol. 96, 723–730. doi: 10.1037/0022-0663.96.4.723

[ref17] Falout J. Maruyama M. (2004). A comparative study of proficiency and learner demotivation. Lang. Teach. 28, 3–9.

[ref18] Fang C. Huang B. (2018). The impact of family human capital investment on children's academic achievement. J. Anhui Norm. Univ. 46, 116–124. doi: 10.14182/j.cnki.j.anu.2018.02.016

[ref19] Feng L. (2011). The Relationship between Parental Companionship and Primary School Students' Self-Awareness and Academic Performance (Master's thesis). CNKI Master Thesis Library.

[ref20] Fuligni A. J. (1997). The academic achievement of adolescents from immigrant families: the roles of family background, attitudes, and behavior. Child Dev. 68, 351–363. PMID: 9180006 10.1111/j.1467-8624.1997.tb01944.x

[ref21] Goldenberg C. Rueda R. S. August D. (2008). “Sociocultural contexts and literacy development” in Developing Reading and Writing in Second-Language Learners. eds. August D. Shanahan T. (New York: Routledge), 95–129.

[ref22] Ham E. H. Bitna P. Yoo J. E. Soonbo K. (2020). The effects of parent-child difference in perception of parental supports on academic achievement and self-concept: an application of latent growth modeling. Korean J. Educ. Res. 58, 33–60. doi: 10.30916/KERA.58.1.33

[ref23] Jeynes W. H. (2010). The salience of the subtle aspects of parental involvement and encouraging that involvement: implications for school-based programs. Teach. Coll. Rec. 112, 747–774. doi: 10.1177/016146811011200311

[ref24] Jung E. Zhang Y. (2016). Parental involvement, children’s aspirations, and achievement in new immigrant families. J. Educ. Res. 109, 333–350. doi: 10.1080/00220671.2014.959112

[ref25] Kim D. Lee S. (2019). The effects of self-efficacy, self-regulation, and social support on English achievement. Engl. Lang. Teach. 31, 23–43.

[ref26] Krashen S. D. (1981). Second Language Acquisition and Second Language Learning. Oxford: Pergamon Press, 31–32.

[ref27] Marsh H. W. Craven R. G. (2006). Reciprocal effects of self-concept and performance from a multidimensional perspective: beyond seductive pleasure and unidimensional perspectives. Perspect. Psychol. Sci. 1, 133–163. doi: 10.1111/j.1745-6916.2006.00010.x, PMID: 26151468

[ref28] Maslow A.H. (1987). Theory of Human Motivation. Hong Kong: Huaxia Publishing House.

[ref29] Morris A. Lafontaine M. Pichette F. de Serres L. (2013). Affective variables, parental involvement and competence among South Korean high school learners of English. Stud. Second Lang. Learn. Teach. 3, 13–45. doi: 10.14746/ssllt.2013.3.1.2

[ref30] Nikolov M. (1999). A study of Hungarian Children’s foreign language learning motivation. Lang. Teach. Res. 3, 33–56. doi: 10.1177/136216889900300103

[ref31] Pekrun R. (1992). Kognition und emotion in studienbezogenen Lern- und Leistungssituationen: explorative Analysen [cognition and emotion in academic situations of learning and achievement: exploratory analyses]. Unterrichtswissenschaft 20, 308–324.

[ref32] Pekrun R. Thomas G. Wolfram T. Perry R. P. (2010). Academic emotions in Students' self-regulated learning and achievement: a program of qualitative and quantitative research. Educ. Psychol. 37, 91–105. doi: 10.1207/S15326985EP3702_4

[ref33] Percy M. M. Martin-Beltran M. Daniel S. M. (2013). Learning together: creating a community of practice to support English language learner literacy, language. Cult. Curr. 26, 284–299. doi: 10.1080/07908318.2013.849720

[ref34] Régner I. Loose F. Dumas F. (2009). Students' perceptions of parental and teacher academic involvement: consequences on achievement goals. Eur. J. Psychol. Educ. 24, 263–277. doi: 10.1007/BF03173016

[ref35] Rumbaut R. G. (1994). The crucible within: ethnic identity, self-esteem and segmented assimilation among children of immigrants. Int. Migr. Rev. 28, 748–794. doi: 10.1177/019791839402800407

[ref36] Ryan R. M. V. Lynch J. (1989). Emotional autonomy versus detachment: revisiting the vicissitudes of adolescence and young adulthood. Child Dev. 60, 340–356. doi: 10.2307/1130981, PMID: 2924656

[ref37] Sadikoglu S. Oktay S. (2018). Learning strategies of students studying Russian as a second foreign language, with relation to English as their first foreign language. Qual. Quant. 52, 2101–2109. doi: 10.1007/s11135-017-0623-3

[ref38] Semaan G. Yamazaki K. (2015). The relationship between global competence and language learning motivation: an empirical study in critical language classrooms. Foreign Lang. Ann. 48, 511–520. doi: 10.1111/flan.12146

[ref39] Shih S. S. (2005). Taiwanese sixth graders' achievement goals and their motivation, strategy use, and grades: an examination of the multiple goal perspective. Elem. Sch. J. 106, 39–58. doi: 10.1086/496906

[ref40] Shin D. S. Seger W. (2016). Web 2.0 technologies and parent involvement of ELL students: an ecological perspective. Urban Rev. 48, 311–332. doi: 10.1007/s11256-016-0356-y

[ref41] Suárez-Orozco C. Suárez-Orozco M. (2001). Children of Immigration. Cambridge, MA: Harvard University Press.

[ref42] Wen Z. L. Ye B. J. (2014). Mediating effect analysis: methodology and model development. Adv. Psychol. Sci. 22, 731–745. doi: 10.3724/SP.J.1042.2014.00731

[ref43] Xie G. Wang X. A. (2022). An emotional analysis method for the analysis of cognitive and psychological factors in the change of second language learning model of Chinese mainland students in the post-epidemic era. Front. Psychol. 13:819855. doi: 10.3389/fpsyg.2022.819855 35992486 PMC9384866

